# Embodied cognitive evolution and the limits of convergence

**DOI:** 10.1098/rstb.2024.0255

**Published:** 2025-06-26

**Authors:** Robert Barton, Louise Barrett

**Affiliations:** ^1^ Evolutionary Anthropology Research Group, Durham University, Durham, UK; ^2^ Department of Psychology, University of Lethbridge, Lethbridge, Alberta, Canada

**Keywords:** cognitive evolution, sensory-motor systems, embodiment, cognitivism, brain evolution

## Abstract

Comparative psychology seems to be perpetually bogged down in intractable debates about which species have what cognitive capacities, which criteria to use and whether or not the capacities are domain general. The problem arises from lack of conceptual clarity about how to define, measure and compare cognitive capacities. In turn, conceptual vagueness arises from the use of anthropocentric folk-psychological concepts given apparent scientific legitimacy by framing them in cognitivist, computational terms. This ‘cognitivist gambit’ assumes that cognitive processes necessarily involve representations that are independent of the sensory-motor specializations associated with different body plans and ecological niches. We argue instead that sensory-motor adaptations are not inconvenient confounding variables that should be controlled to isolate cognition, but intrinsic aspects of cognitive evolution. This perspective implies that, because bodies and their sensory-motor control are highly divergent across the tree of life, comparative psychology should pay more attention to phylogenetic constraint and divergent cognitive evolution. It also implies that boiling down neuro-cognitive evolution to brain size or numbers of neurons will fail to capture the richness and complexity of the interrelationships between nervous systems, cognition, behaviour and ecology. If correct, this perspective suggests a need to reconsider the ontological basis of comparative psychology.

This article is part of the Theo Murphy meeting issue ‘Selection shapes diverse animal minds’.

## 1. Introduction

Do non-human species recall past events, plan for the future, possess a self-concept or understand the mental states of others? These are among the many questions that comparative psychology has wrestled with for many years, and for which clear and widely accepted answers are still lacking. For example, we know that animals behave in ways that, on some level, anticipate future needs and rewards: migratory birds plot courses across thousands of miles to breed or winter in more propitious circumstances [1], female mammals wean their offspring in readiness to produce the next litter [2] and New Caledonian crows select tools they will need in the future [3]. A variety of species, including—to the surprise of some—fish, can orient towards and use their reflections to guide self-directed behaviour [4–7]. Rats aid conspecifics in distress [8], monkeys respond to predator-specific vocalizations by showing predator-specific escape behaviours [9] and great apes anticipate where actors with a false belief about the location of something will search for it [10]. At a behavioural level, therefore, animals appear to use information to guide their behaviour towards adaptive outcomes in quite complicated ways. Comparative psychologists, rightly, want to know how they do it; in particular, are simple ‘low-level’ explanations sufficient, or is ‘cognition’ involved [11]?^1^

Typically, by ‘cognition’ comparative psychologists mean that an animal generates internal *representations* that can stand in for stimuli in their absence, and which can be manipulated and transformed to find the solution to a given problem [14–16]. Indeed, when asked to define cognition, most researchers explicitly mentioned the importance of mental representation [17]. For example, ‘taking the cognitive approach involves asking what information is (in some way) represented by an individual: what it notices, remembers, and can perhaps compute with’ [17].

Here is the difficulty: there is little agreement on the criteria necessary to demonstrate the existence and use of such internal representations. Ingenious experiments have shown, for example, that scrub jays adjust their foraging behaviour according to multiple sources of information in ways that are consistent with the formation and manipulation of internal representations of what happened, where and when [18]. Rats also solve problems in ways that are consistent with the possession of a capacity to represent past events [19]. Explanations that are only *consistent* with a particular interpretation, however, do not (indeed, cannot) rule out plausible alternatives [20], leading to unresolved debate [21,22] and the guarded use of agnostic terms, such as episodic-*like* memory [18,22]. Similarly, there is ongoing debate and disagreement over whether experiments on tool selection in New Caledonian crows reveal an ability to represent their future needs [23,24]. Although researchers are avowedly keen to distinguish between cognition and supposedly simpler mechanisms, there is surprising variability in what this is taken to mean and therefore how it should be done [25]. Debate over the interpretation of data is normal in science. In general, however, we do expect to see gradual progress towards a consensus on accepted facts and how to demonstrate these and rule out alternative explanations, in a way that remains hard to discern in the field of comparative cognition [11,22,26–29], even leading some to a counsel of despair [28].

What is behind the lack of clear progress?^2^ Perhaps the very formulation of the questions makes them inherently insoluble: how can one demonstrate the existence of a phenomenon (i.e. a cognitive representation) for which alternative explanations are seemingly impossible to rule out? The solution to this conundrum is by no means obvious (e.g. [26,31–33]) and for this reason, the goalposts for demonstrating a particular representational capacity seem to be perpetually in motion (e.g. [21,28,34]) . This is one though not the only reason behind the suggestion that postulating representations does no useful conceptual or epistemic work and that the field of animal cognition should seek explanations that do not invoke them [35–38].

Allied to the idea of ‘cognition as representation’ is the notion that cognition is distinct from the ‘run-of-the mill’ sensory-motor processes that govern interactions with the environment [17,39–41]. That is, in a cognitive information-processing paradigm, sensory and motor abilities are seen as functioning as peripherals, concerned only with the input to and output from the computational processes occurring in the decision-making parts of the brain. In principle, similar computations could be performed on inputs of different kinds and used to inform the decision-making parts of the mind about the appropriate motor outputs. As such, the specific nature of the sensory and motor capacities possessed by different species is seen merely as inconvenient confounding variables for evaluating differences in cognition. There is thus an implicit assumption that a focus on representational processes crucially captures some essential quality of brains not tied to any one species, but applicable to brains in general. Consequently, cognitive evolution can, in principle, be separated from the rest of the biology of the animal, such that abstract representational capacities and the computations on which they depend can evolve convergently among very different species on widely separate branches of the tree of life [42–44]. In turn, these representational capacities enable ‘executive control’ of behavioural outputs: they are ‘domain-general’ because they can be implemented in a variety of different systems rather than depending on specific types of information associated with solving specific types of behavioural problems [45].

Yet, this view of cognitive evolution as the evolution of representational capacities distinct from sensory-motor control faces serious problems. Notwithstanding the already noted difficulty of convincingly supporting or falsifying representational hypotheses and the consequent lack of agreement about whether species X has capacity Y, cognitive representations may not be separable in the way imagined. As Ölveczky in Bayne *et al.* [17] says ‘neural circuits do not implement “cognition” or other vague concepts inherited from philosophy and psychology’. The box-and-arrow flow charts traditionally used by cognitive psychologists to portray transitions between inputs, cognitive manipulations and outputs, do not exist in the brain. For example, it is conventional to refer to the visual cortex of the primate brain [46], and this is usually identified with brain areas early in the processing hierarchy as distinct from ‘association’ regions of cortex assumed to do the cognitive work [47]. Since the primary visual cortex (area V1) is proportionately small in larger primate brains, because other cortical areas show greater proportional expansion, visual processing is generally not greatly considered in accounts of brain evolution and is entirely disregarded in most accounts of primate cognitive evolution. However, visual projections ramify throughout the cortex, including not only occipital but temporal, parietal and frontal regions [46,48,49]. This is very likely why the relative expansion of supposedly ‘non-visual’ cortex correlates strongly with variation in visual inputs, subcortical visual nuclei and area V1 [50,51]. In fact, a recent analysis by Meyer *et al*. [49] demonstrated that the further along the processing hierarchy a visual region is found, the greater its relative size in humans compared with macaques: area V1 in macaques is 51% of the size of human V1 whereas the corresponding figure for extra-striate cortical areas is just 14%. Furthermore, meta-analysis of fMRI data have revealed that these extrastriate areas are involved in spatial attention, visuo-motor guidance, working memory and numerical processing [49]. This raises the thorny issue of which of these functions should be considered visual and which cognitive, and what determines this demarcation. Where is the dividing line in the brain between visual perception and cognition?

Perhaps such questions make no sense. Perhaps we should abandon the search for ‘cognition’ in terms of representational capacities distinct from systems for sensing and moving, and instead view cognitive evolution as inextricably linked with the mechanisms that animals use to guide and control their actions and interactions with their worlds. In what follows, we develop this argument and explore its implications for understanding cognitive evolution and its neural basis. We start by describing an unresolved tension in comparative psychology between the concept of evolutionary convergence and the general framework imported from human cognitive science, which we term the cognitivist framework. We argue that this framework draws the study of cognitive evolution ineluctably towards both anthropocentric thinking and an untenable dualism between cognition and sensory-motor control. Not only is this dualism untenable, it impoverishes the study of cognitive evolution by ignoring the rich diversity of mechanisms involved in animal behaviour. It also impoverishes the study of neurocognitive evolution by encouraging the search for misleadingly simple neuroanatomical metrics of supposedly unidimensional cognitive phenomena, like intelligence. The importing of this framework from cognitive science is a gambit that has not paid off.

## 2. Convergence and the cognitivist gambit in comparative psychology

The dominant paradigm in comparative psychology makes a set of interrelated assumptions about the nature of cognitive evolution. Specifically, the capacities that have evolved are generalized capacities enabling behavioural flexibility, executive control, insight, innovation and problem solving; ‘intelligence’, broadly conceived (e.g. [42,45,52,53]). These capacities are considered to vary quantitatively and continuously across the tree of life and to have evolved convergently under similar selection pressures in different lineages [54–58]. This notion of evolutionary convergence implies, first, that the same or similar cognitive processes can be mediated by very different brains. Second, widely divergent taxa on different parts of the tree of life have evolved the same general cognitive solutions to similar problems [43,44,54,59], much like the convergent evolution of wings as a solution to the adaptive challenges of flight [54]. Although many authors highlight specific cognitive capacities, such as social intelligence, metacognition and inhibitory control, recently these have been conceived of as elements of a unified ‘General Intelligence’, with explicit parallels drawn between individual differences in intelligence in humans and variation in general intelligence across species [45,52,53,60,61].

There is a contradiction within this paradigm, however. Despite the emphasis on convergence in cognitive capacities, epithets such as ‘higher’, ‘advanced’ and ‘sophisticated’ are routinely attached to them, implying an unresolved tension between notions of convergence and of evolutionary hierarchy. The tendency for comparative psychology to portray cognitive evolution in anthropocentric terms, as a *scala naturae*, has been critiqued repeatedly, and for many years (e.g. [16,40,62,63]), yet it remains embedded and implicit in such terminology and hence in the concepts used in designing research. Within the broader field of evolutionary biology, this appears to be a particular problem in the study of cognition, as such terms are rarely attached to non-cognitive traits: we do not generally find reference in the literature to higher locomotion, advanced life histories or sophisticated metabolism. The reason is likely to be that comparative psychology has drawn many of its concepts from a largely evolutionarily uninformed human psychology, whereas evolutionary biology thinks phylogenetically and ecologically. While we accept that this is a somewhat crude characterization of a more diverse field that includes the traditions of cognitive ethology and behavioural ecology, it seems that the study of cognitive evolution is nevertheless still strongly influenced by anthropocentric concepts.

It might be thought that emphasis on convergent evolution is antithetical to anthropocentrism, because it allows for the emergence of ‘sophisticated’ cognition in phylogenetic branches far removed from our own [43]. This is only partly true, however, because the cognitive traits that researchers look for in other species are derived from anthropocentric notions of what cognition entails [63], notably ‘intelligence’ and its components. And this lures researchers—possibly unconsciously—onto the *scala naturae*. If we were to take the notion of evolutionary convergence seriously, whether crows are ‘feathered apes’ [43], for example, ought equally to invite the question of whether humans and other apes are wingless crows, limbed dolphins or bony octopuses. That the question is never posed in these ways suggests to us an underlying anthropocentrism.

The *scala naturae* is thus often hidden beneath the surface of theoretical approaches in comparative psychology, but persistently resurfaces to influence empirical work. When considering how psychological traits vary across the animal kingdom, they are frequently described as extending from so-called ‘simple reflexes to sophisticated intelligence’ [44]; a perspective that can be traced back to the origins of comparative psychology, at least as far as Romanes:

The lower down we go in the animal kingdom, the more we observe reflex action, or non-mental adjustment, to predominate over volitional action … the less capacity do we find for changing adjustive movements in correspondence with changed conditions. [64]

More than 130 years later, aspects of Romanes’ conceptual framework for studying species differences in cognition remain embedded in the field. His notion of variation in the capacity for adjustive movements corresponds closely to research in comparative psychology focused on mechanisms of reasoning and executive or inhibitory control [56,60]. As Smith [65] and Parvizi [66] have described in detail, such concepts have an interesting cultural history associated with Western anxieties about the maintenance of moral order and the control of society. These concepts became embedded in psychology and neuroscience, and hence into the computational approach to mind. Cognition became seen as a set of control processes inhibiting instinctive responses, thereby enabling rationality. It is unclear, however, that it is safe to assume that these culture-bound concepts are suitable for understanding the evolution of animal minds, or indeed even the human mind specifically [67–69].

## 3. Anthropocentrism and computationalism

Traits studied by comparative psychologists include reasoning, causal inference, executive control, working memory and planning. Such traits are central to the classical computational theory of mind that arose following the cognitive revolution (see [70,71]); the aim was to model (or even recreate) human intelligence through the use of computers. Consequently, ‘higher’ or ‘advanced’ cognitive traits always translate into those that humans are known to possess [17,35,72]. This computational framework was adopted by researchers in comparative cognition because it seemed to offer a clear alternative to behaviourist approaches, which were seen as excluding internal processes from consideration (although many of these did no such thing [71]). Focusing on internal processes rather than behaviour alone was viewed as restoring the proper subject matter of psychology, the mind, to its rightful place. In doing so, the anthropocentric origins of the computational framework, and the cognitivist position more generally, went unrecognized, forgotten and ignored.

Although it might be argued that a cognitive representational approach is not inherently anthropocentric, this argument is difficult to defend. As Brooks [70] observed, even after the pioneers of the cognitive revolution recognized the intractability of recreating human intelligence in a machine, the more circumscribed set of tasks on which they then focused were still ‘bench-marked against the sorts of tasks humans do within those areas’ [70, p. 14], such as symbolic algebra, geometrical problems and natural language understanding. Moreover, the researchers themselves defined and refined the problem domains, abstracting away most details to create simplified task descriptions and thereby reducing problem complexity. Brooks [70] points out that this meant humans had already accomplished most of the intelligence needed to solve the task—specifically the abstraction process—before artificial systems were even involved. This abstraction process thus ensured that representational-computational theories remained anthropocentrically oriented: first, and most obviously, because humans had predetermined the relevant computations that would be needed, but also because the abstraction process was assumed to generate computational programs that experienced the perceptual world in the same way as humans. Recognition of this latter point in particular has been lost, because of the way perceptual processes are considered to serve only as inputs and play no role in ‘cognition proper’ [70,73]. Hence, when claims about the convergent evolution of intelligence are made, they are inevitably cast in anthropocentric terms, because they arise from a theoretical framework devised to characterize human capacities and these are modelled in computational terms that inherently reflect human capacities and concerns. As such, a cognitivist approach implicitly embeds a notion of a *scala naturae* into our assessment of other species’ cognitive capacities. Other species are implicitly cast as ‘duller, less talkative versions’ of ourselves [74, p. 29] and the twin influences of computationalism and anthropocentrism come to shape the aims of comparative psychology.

Note that an interest in human evolution and human uniqueness does not justify using anthropocentric cognitive concepts in comparative psychology. First, what makes humans unusual or unique is unlikely to be what—in varying quantities—distinguishes one non-human species from another. Why should it be, if we do not subscribe to a notion of evolution as progress towards humanity? It is possible, therefore, that we will never learn anything truly meaningful about cognitive evolution in general if we continue to employ such concepts. The second point follows from the first; continued adherence to anthropocentric concepts will actively hinder our ability to define human uniqueness, as this can be achieved only if we have a broader understanding of the patterns and processes of cognitive evolution across the tree of life.

However, the problems with the computational approach go beyond anthropocentrism. The idea of cognition as the manipulation of representations, independent from sensory-motor control, leads researchers to ignore the possible ways in which the two things are related. For example:

A common assumption of cognitive scientists and behavioural biologists is that perception involves the ordering of sensory information into internal representations, and that cognition involves the manipulation of representations (derived from both perception and memory). If this is so, then defining cognitive complexity could, in principle, be quite straightforward. Organisms construct internal (cognitive) models of the world and cognitive complexity is just the complexity of these models. [40, p. 2680]

We suspect that things will not prove to be so straightforward. One issue is that complexity is usually not operationalized, rather it is left unhelpfully vague. This leads to a second problem: assessment of the complexity of an organism’s cognitive models must, by necessity, be made by inference from the complexity of behaviour (given that such internal models cannot be observed directly), but there is no necessary link between the complexity of a behaviour and the complexity of the mechanisms that underlie it (see Barrett [75,76] for examples). Third, the notion of cognitive representations creates the problem of infinite regress, wherein the representation must be re-presented for scrutiny by some higher-order cognitive entity in order to be translated into, or influence, action, a problem that has been described as homunculi-inside-homunculi [38]. The problem we wish to primarily focus on here, however, is the related one that we have pointed to above: the very concrete difficulty of distinguishing between perception, or sensory-motor control more broadly, and cognition. This distinction may seem logical to many because of the deeply embedded allure of dualism. If cognition can be construed as a set of representational processes independent of adaptive dynamic, sensory-guided control of the body,^3^ then cognitive evolution can be studied independently of the specific biological features of phylogenetically distant species; that is, we can safely ignore bodily adaptations (grasping hands, wings, beaks, eyes, sonar, tentacles, etc.), their interrelationships with nervous systems and how they mediate the animal’s interactions with its environment.

Comparative psychology in the main, therefore, subscribes—implicitly if not explicitly—to an evolutionary form of what is known in philosophy of mind as functionalism [77]; the same cognitive processes can in principle be implemented (or ‘realized’) by different physical systems, whether artificial or natural and whether in apes or octopuses. Just as flight can be carried out using feathered or membranous wings, cognitive processes such as Theory of Mind and episodic memory can be carried out by differently organized brains. If one is trying to isolate cognitive from sensory-motor control, it does not matter if, like an octopus, you have a soft body with infinite degrees of freedom enabling three-dimensional movement in the medium of water, or a jointed skeleton, like humans, adapted for walking in two dimensions on land and manipulating the world with hands: cognition is a generalized capacity that can be untethered from such ‘constraints’. Species differences, in an important sense, are therefore treated as ‘species neutral’ because cognition itself is conceived as a species-neutral, disembodied capacity [78] functioning independently of its sensory inputs and behavioural outputs. Working in such a framework, different species can be studied using the same tests, or analogues of the same tests, that have been adjusted to account for sensory and motor differences and designed to get at fundamentally the same representational cognitive processes.^4^ This assumption—that we can bracket off and control for differences in the mechanisms of sensory-motor control in such a way that we can isolate ‘purely cognitive’ capacities [40,80] and study them in diverse organisms—is problematic. As Godfrey-Smith [81, p. 270] points out,

minds exist in patterns of activity, but those patterns are a lot less ‘portable’ than people often suppose; they are tied to a particular kind of physical and biological basis.

We suggest that this mental non-portability across species arises because the evolution of neurocognitive systems is inextricably bound up with the ways in which animals sense, move through, explore, learn about and manipulate their worlds.

## 4. The neural basis of cognitive evolution

There are clear implications of these arguments for how we study the neural basis of cognitive evolution. Allied to the popular idea that domain-general intelligence involves computational capacities that have evolved convergently, and at the same time reflecting the uneasy tension with notions of evolutionary progress, neural mechanisms of cognitive evolution similarly are construed as involving ‘cognitive’, ‘higher’, ‘advanced’ or ‘more recently evolved’ (versus ‘primitive’) parts of the brain: that is, regions that have expanded during evolution, and have done so convergently in distinct lineages selected for intelligence [82]. In mammals, this is held to be the neocortex or particular neocortical regions, in birds the pallium and in octopuses the vertical lobes [55,82–84]. In frankly *scala naturae* terms, Rakic [83, p. 724] even describes the neocortex as ‘the crowning achievement of evolution and the biological substrate of human mental prowess’. These ‘cognitive’ brain structures are viewed as modality-independent information-processing, associative computational devices (‘onboard computers’), whose cognitive operations can be detached from sensory-motor and affective processes. They are argued to vary among species principally in their overall computational capacity, as reflected in measures such as volume or numbers of neurons [45,52,84,85], and are considered capable of evolving and expanding convergently to support ‘advanced cognition’ in different groups of animals such as mammals, birds and cephalopods [55,85,86]. Because these structures make up a large proportion of the brain, their size tends to correlate with overall brain size. Accordingly, simple and easily obtained neuroanatomical measures such as brain size, neocortex or pallium size and numbers of neurons are seen as useful proxies for intelligence, sometimes anthropocentrically validated by the observation that humans have the largest brains (relative to body size), the most neurons and purportedly the greatest quantity of intelligence [84].

Although this stance seemingly ensures comparisons will be straightforward and empirically tractable, there has been prolonged and continuing debate regarding which measures provide the best measure of its mechanistic implementation across species, whether or not and how to control for the effects of body size, and at what taxonomic level valid comparisons can be made in the face of differences in brain organization [84,87–89]. This ultimately has led to a confusing picture and no clear consensus [90–93]. To anticipate our conclusion on this, we consider the whole debate as misguided, because intelligence—conceived as species-neutral, portable computational capacity—is not a useful comparative concept and simple neuroanatomical metrics cannot capture the gloriously messy and complicated reality of neuro-cognitive evolution [91–94]. Debates of this nature are a bit like arguing whether the size of an engine, or the volume or number of its cylinders and whether or not to control for the weight of the car, offers the best measure to assess the design and functioning of the engine. Engine size and number of cylinders may well correlate with certain behavioural measures such as acceleration, but focusing on acceleration as the most interesting trait seems an impoverished approach to understanding how engines work and how and why they vary. The greater the diversity of the objects of study (vehicles or species) the more true this will be (for example, if we wish to consider engines designed for aerial versus terrestrial propulsion). The metaphor is itself imperfect, however, and the situation is much worse than this: speed and acceleration are real properties that can be measured objectively and compared directly between types of vehicle. Intelligence is not like that; it cannot be measured directly because it is an abstraction based on culture-bound and anthropocentric assumptions about the nature of cognition across species.

We do not argue that brain size evolution is devoid of interest: brain-size variation is substantial, interesting, phylogenetically patterned (e.g. [95]) and demands explanations, in terms of which neural systems contributed to it, how it relates to body size and what constraints have operated. The problem arises when we treat brain size as an explanation in itself and hope that it corresponds to some unitary cognitive attribute, acting as a potential neural metric of ‘intelligence’. Adequate explanations for brain-size variation, however, are unlikely to be as simple as intelligence or its pseudo-theoretical computational synonym ‘processing power’. They are likely to be complicated and multivariate, reflecting the fact that different adaptive changes occurred at different times on different parts of the tree of life, involving a host of constraints and diverse neural and sensory-motor specializations not usefully reduceable to a single comparative dimension of intelligence [92,93,96].

The problem is not solved by isolating specific brain regions as the locus of an animal’s intelligence. A fundamental problem with the attempt to separate ‘cognitive’ from ‘non-cognitive’ brain regions is that this distinction does not correspond at all to the way that brains are organized, the way they evolve or the processes they support. In line with the cognitivist gambit, ‘cognitive’ regions are conceived as functionally and anatomically distinct from the sensory-motor systems that directly control interactions with the environment [40], encouraging a dualist narrative in which cognition floats free, and varies independently, of supposedly more basic processes mediated by supposedly more primitive brain structures. This framing therefore privileges brain areas, structures or cortical regions (such as prefrontal cortex), over distributed networks, running counter to the fact that all behaviour depends on multiple brain regions. In mammals, cortical and non-cortical regions function and evolve in highly integrated ways to mediate adaptive behaviour [97–99], such that ‘cognitive function cannot be assigned to either the cortical or the sub-cortical component, but instead emerges from their tight co-ordination’ [98, p. 2559] . The same applies to distinct cortical areas. These facts must have important implications for understanding the neural bases of cognitive evolution and for how we evaluate them.

Corresponding with network-based views of brain function, mammalian brain evolution, often simplistically characterized as the progressive enlargement and elaboration of the neocortex [83,100] in fact involved coordinated change in networks connecting cortical and sub-cortical brain regions, with anatomical connectivity across major brain regions being a strong predictor of the correlated evolution of those regions [92,97,101–103]. There is still latitude for major regions to show some independent evolution [101], but this is as likely to involve supposedly ‘lower’ brain regions as supposedly ‘higher’ regions; natural selection does not recognize this distinction. To give one example, contrary to the assumption that neocortical expansion was the neural substrate of cognitive evolution in primates, apes deviate significantly from the general pattern of tightly correlated evolution between neocortex and cerebellum because the cerebellum expanded rapidly relative to the neocortex, across multiple hominoid phylogenetic branches [104]. Despite these facts, the twin biases of corticocentrism and anthropocentricism continue to bedevil evolutionary perspectives on mammalian brain structure and function [97–99,105]. This state of affairs is driven in part by the use in cognitive neuroscience of concepts drawn from psychology, despite the fact that these were conceived in the 19th century as ways to explain human cognition without regard for biology or evolution [106]. Pessoa *et al*. [107, p. 3], go so far as to suggest that traditional psychological concepts, or ‘mental terms’, are epistemically sterile … ‘vertebrate neuroarchitecture does not respect the boundaries of mental terms’. If correct, this presents a problem for approaches to comparative psychology and brain evolution which draw their terms and concepts from the same tradition. It suggests we need to rethink our ontological assumptions.

A clear statement of the position that cognitive and sensory-motor adaptations can be treated as independent is provided by Triki *et al*. [39]. These authors make two claims. The first is that much of the variation in brain size across species and major clades can be understood in terms of the evolution of sensory-motor systems. This is a claim with which we agree, and together with others we have provided empirical evidence and theoretical arguments to support it [50,51,76,96,97,108–111]. Triki *et al*. [39] interestingly explore possible ecological explanations for major clade differences in the elaboration of sensory-motor systems, in particular, the effects of shifts between aquatic, terrestrial and aerial niches (see also [96]). Where we differ is on their second claim, which is that fish, reptiles, birds and mammals may have similar amounts of cognition but differ greatly in their sensory-motor mechanisms, and hence brain size (see Fig 1 in Triki *et al.*, which shows different proportions of the brain devoted to general intelligence, cognitive toolkits, memory and sensory-motor systems). This idea is reminiscent of MacPhail [80], who argued that, once ‘contextual abilities’, including sensory-motor skills, were controlled for, there was insufficient evidence to reject the null hypothesis of no species differences in intelligence (excepting humans). We argue that the anticipated programme of research to separate cognitive and sensory-motor skills is a wrong turn, because cognitive and sensory-motor adaptations are not separable in the way envisaged.

We propose instead that attention should be paid to the divergent, embodied nature of cognitive adaptations, with sensory-motor specializations incorporated as an intrinsic aspect of cognitive evolution. This has the corollary that, instead of boiling down ‘endless forms most beautiful and most wonderful” [112, p. 490] to unidimensional notions of cognitive advancement, and its neural basis to brain size, appreciating the ways in which cognitive evolution is embodied and intrinsically connected to the diverse sensory-motor and affective mechanisms of behaviour will enrich our understanding of the variegated ways in which behavioural complexity has been achieved across the tree of life. This is not new; it is in essence, what many neuroethologists have already been doing for some time (e.g. [113]) and work continues in this vein [96,110]. Yet, such work has become isolated from comparative psychology and the study of the macroevolution of brain, behaviour and cognition [93]. We suspect this is because phenomena such as bat echolocation [114], primate visuo-motor specializations [48,50,51] and butterfly visual learning [110] are usually considered irrelevant to cognitive evolution. In part, therefore, our plea is to reconnect neuroethology and comparative psychology.

## 5. What is it like to be an octopus?

Thomas Nagel [115] famously argued that we cannot know what it is like to be a bat, because bats live in an entirely different sensory-motor world to that of humans. Projecting our own consciousness into the body of a bat will not do, for that simply concocts a human idea of what it would be like for us to hang upside down in a dark loft by day and fly about catching insects in the dark, as though the fundamental properties of bat consciousness might be, in essence, no different to our own once we have accounted for our different sensory worlds. Sonar-guided flight and foraging is intrinsic, however, to how a bat might feel and think. We make a related point about understanding cognitive evolution: the precise nature of an embodied biological system—that is, of an animal, taken as a whole in its ecological context—determines the manner in which it explores and learns about its world, and that in turn determines what and *how* it knows about it. As such, projecting properties of our own minds into the bodies and brains of other species, albeit in lesser quantities, will not be informative. But this is frequently what happens. Cephalopods form a case in point.

Cephalopods intrigue comparative psychologists, because they have evolved large and complex nervous systems and flexible behaviour, problem-solving abilities and tool use completely independently of vertebrates and with very differently structured nervous systems (e.g. [44,86,116,117]). If, as comparative psychologists have suggested, intelligence evolves convergently, cephalopods should present an instructive example: to what extent can similar selection pressures result in similar intelligence? The answer in this case, it seems, is not much. Beyond the superficial similarity that cephalopods have elaborate nervous systems and large brains, unlike large-brained vertebrates, they are mainly solitary and comparatively short-lived [44,58]. Moreover, brain size does not correlate with sociality or life history in this clade [86]. The distinctive socio-ecological and life history contexts of cephalopod neuro-cognitive evolution ought perhaps to make us pause before applying the usual interpretative frameworks.

In their review of cephalopod cognition, Amodio *et al.* [44] acknowledge the very different bodies and nervous systems of cephalopods and recognize the notion of embodied cognition. They then, however, focus their discussion on precisely the same species-neutral capacities that form the mainstay of comparative psychology, emphasizing their convergent evolution with vertebrates in respect of their flexible behaviour and large brains, and enquiring whether octopuses, like humans, have a Theory of Mind. Octopuses are indeed extremely flexible, in terms of both their bodies and their behaviour, yet despite their pliant characteristics we cannot just squeeze them into existing theoretical frameworks and hope they will fit comfortably. We need to take their bodily flexibility and its distinctive mechanisms of sensory-motor control—and the radical differences in the way that they and vertebrates are organized—much more seriously if we are to understand the nature of their cognition and the mechanisms mediating it [118,119].

More broadly, we may perceive similarities between cephalopods and vertebrates in their behavioural patterns and abilities to solve problems, without assuming that they are governed by similar processes. Accordingly, it may be unproblematic to refer to intelligent *behaviours* in phylogenetically divergent species, so long as we do not slip from the description of a behaviour as intelligent to the assumption that a similar form of cognitive process is the explanation. The differences are surely at least as interesting and informative as the similarities. The soft, unjointed bodies of octopuses have near infinite degrees of freedom to execute movement, and this body plan is associated with a nervous system with both centralized and distributed control [116]. Motor regions of the central brain lack the somatotopic mapping found in mammal brains [120,121]. Most neurons are found in ganglia in the arms, and the arms are endowed with a unique chemo-tactile system derived from secondarily adapted neurotransmitters for contact-dependent chemosenzation [122]. Distributed neural control means that amputated octopus arms continue to exhibit complex exploratory behaviour. Carls-Diamante [119] suggests that this raises the question not only of *what* it is like to be an octopus, but *where* it is like to be an octopus. While that question raises a different problem, that consciousness may not be locatable in the way implied [123], we do not wish to get sidetracked here into such phenomenological questions. Our point is that appreciating the distinctive nature of octopus bodies, nervous systems and ways of exploring the world will be key to asking the right scientific questions about their cognition. To get inside the mind of an octopus, you would also need to get inside its body [82]. If we refocus our attention on interrogating the ‘pivotal role played by body, sensory, brain and motor traits’ [96], we can frame our scientific questions in more cephalopodocentric (or corvocentric, delphinocentric, etc.) terms.^5^ And we can then understand how it is that animals make adaptive decisions without resorting to problematic, culturally embedded folk-psychological notions like ‘executive control’.

This prescription does not single out cephalopods as requiring special explanations. Although octopuses have become seen as the poster animal for embodied cognition, this rather misses the point. Their cognition is no more and no less ‘embodied’ than that of other creatures; embodiment is a general principle, not a trait. Our prescription is a general one for understanding divergent cognitive evolution associated with divergent sensory-motor worlds. Indeed, once this shift in perspective has been made, it becomes easier to see that the same must apply to mammalian bodies, including our own. For example, in an ingenious experiment, Garcia-Pelegrin *et al.* [125] examined the facility of three primate species to perceive and predict the outcome of sleight-of-hand actions performed by experimenters, finding a significant effect of species differences in thumb opposability. This result is not obviously predicted by a standard cognitivist framework postulating variation in the ability to represent outcomes independent of sensory-motor specializations. It does, however, fit with suggestions about the co-evolution and co-functioning of the human hand and human cognition [126,127]. Another example is work on object permanence in human infants [128]. This has shown that the ability to pass a classic test, the A-not-B task, can be predicted and controlled by changing the physical dynamics of infants’ bodies. For example, a baby that fails the test can be shown to pass if weights are attached to their arms during a certain part of the test, or if they are placed into a standing, rather than sitting, position. Changes to the physical body should not matter if infants have formed a stable, internal representational concept of an object in their brains, which the test is assumed to probe. If instead we consider the infant itself as a dynamic cognitive system, and view the concepts and beliefs, that we frame linguistically as distinct mental states, as distributed over its brain and body (i.e. genuinely regarding the whole baby as the entity that shows object permanence) then these modifications are entirely explicable as actions that help move the system from one stable attractor state to another, so enabling success.

Shettleworth’s [129] definition of cognition as ‘all the ways in which animals take in information through the senses, process, retain and decide to act on it’ is quite routinely quoted by comparative psychologists, yet in our view rarely taken as seriously as it should be. Somewhere between quoting this definition and working through its implications, everything narrows down to a more conservative (*sensu* Heyes [129]) cognitivist interpretation of the decision-making part, as if that floats free of the rest. Thus, even though the visuo-motor specializations of primates have fundamental implications for the ways their brains are structured [130,131] and vary in size [50,51] and for the manner in which they explore and learn about their world, comparative psychology attempts to strip these away to study their cognition. We hope that readers see and grasp our point. Attempting to ‘control for’ such ‘confounding variables’ ignores crucial aspects of how animals navigate their worlds; it is profoundly un-biological and un-ecological.

## 6. Divergence and convergence

The theme issue of which this paper is a part invited authors to consider how natural selection has shaped diverse animal minds. Our general point is that comparative psychology ought to engage more fully with that diversity, rather than attempting to boil cognitive evolution down to how intelligent different species are, how much executive control they have, or the extent to which they share human faculties such as episodic memory. This would entail treating animals more on their own terms as specialists in particular ways of making a living, and engaging with the particularities of their minds in terms of how they sense, explore, learn about and respond to the distinctive features of their worlds.^6^ In turn, this encourages us to explore cognitive evolution as a diversity-producing process as well as a process that may result in convergence, particularly at the level of behaviour. It encourages us to ask question about how diversity in cognitive processes relates to all the aspects of the ‘ways in which animals take in information through the senses, process, retain and decide to act on it’[129], seeing these as elements of a unified process, rather than attempting to separate off the ‘retention and decision’ elements and characterizing them as lesser versions of how we subjectively feel that we do those things as humans, involving all the socio-cultural and linguistic scaffolding that is not relevant to non-human species.

Our rejection of evolutionary functionalism entails a whole-organism approach to cognitive evolution: different nervous systems evolved to guide different bodies using different mechanisms based on different aspects of the sensory environment in the context of different ecologies. In turn, this means that cognitive traits cannot be species-neutral; they all have a distinct phylogenetic history intrinsically associated with the ways bodies and nervous systems have evolved to enable adaptive behaviour across diverse clades. Furthermore, the search for unidimensional metrics of the neuroanatomical basis of intelligence, separate from sensory-motor systems, is an impoverished approach doomed to failure. Neither brains nor specific homunculi-bearing regions of brains are on-board computers into which one could theoretically feed any kind of sensory inputs, or output to any kind of motor system. We have argued that the notion of a separation between sensory, motor and cognitive functions is theoretically flawed and heuristically unproductive. And yet, as we have shown, much of comparative psychology and the related search for the neural bases of cognition depend on such a distinction.

Where does this leave the notion of evolutionary convergence? To be clear, we do not question the idea that convergent evolution is interesting and important, nor that it is useful to study its role in cognitive evolution. Comparative analyses of convergent traits provide powerful evidence for adaptive evolution. We also agree that there are commonalities in the nature of problems that organisms must solve. For example, the need to construct coherent, well-articulated, sometimes complicated sequences of behaviour to obtain rewards, guided by sensory-motor feedback, is a rather general problem [135,136]. How do different species achieve such sequencing, using what cognitive processes and neural mechanisms? What learning processes are involved? To what extent are there non-adjacent dependencies and syntactical structuring [137] in natural, ecologically relevant behaviours [138]? Even more fundamentally, we can point to the convergent appearance of centralized nervous systems associated with motility [139]. It is no coincidence that the three lineages—chordates, cephalopods and arthropods—that independently evolved complex, centralized brains and that have attracted the attention of comparative psychologists, are also the lineages in which we find complex motility [96].^7^ There is, therefore, convergence at the level of behaviour. The neural mechanisms and the cognitive processes will, however, vary [15], so understanding how motile animals produce elaborate sequences of apparently goal-directed actions requires us to delve into the details of the implementation and how these differ. Starting with observations about behavioural convergence, we can then ask detailed questions about cognitive processes and neural mechanisms without needing to invoke the kind of generic cognitive categories, species-neutral computational capacities or abstract representations whose relevance we have queried.

This perspective suggests that, as Coombs & Trestman [96] have advocated, far more attention should be paid to the divergent nature of cognitive adaptations, and how these are rooted in the sensory-motor specializations mediating animals’ interactions with their environments. Convergence and divergence are inherently phylogenetic concepts, in that they imply divergence from an ancestral state followed by convergence towards derived, homoplastic states, which can only be understood within a historical phylogenetic framework. This is sometimes easiest in comparisons of relatively closely related species, as in the elegant work by Hodge *et al.* [110] linking visual specialization, memory, foraging behaviour, their neural substrates and ecological niche in *Heliconius* butterflies. Yet, an explicit phylogenetic framework and understanding of the mechanisms involved is often lacking from discussions of cognitive convergence. In order to understand the true nature and extent of convergence, a more thoroughgoing phylogenetic perspective is needed in which divergent mechanisms and behaviours provide an interpretive context for convergence.^8^ Put another way, convergence can only be understood within a framework that incorporates divergence—otherwise, there can be no clarity about the nature of the convergence, from and to what states, or about its limits. This also entails a more biologically and ecologically grounded analysis in which neuro-cognitive processes and their evolution are intrinsically related to phylogenetic history, how animals explore and learn about their worlds using sensory-motor adaptations. In short, a biologically plausible account of cognitive evolution requires us to understand the whole animal in its world and in its phylogenetic context. This is rather different than trying to understand cognition as a generic computational device on legs—or on wings or fins or tentacles.

Given that, in our view, cognition is inherently embodied, the term ‘embodied cognition’ is tautologous. It is, however, a necessary tautology for as long as psychology continues to be influenced by the notion that cognitive processes could, in fact, be disembodied. The term embodied cognition has come to have several meanings, and we are not sympathetic to them all; we use it here specifically in the sense that centralized, allegedly abstract, cognitive processes are built from sensory-motor functions [141]. Although ‘off-line’ cognition, when we sit and think, can occur without simultaneous action, even this is body-based, activating the same systems used to guide and predict outcomes of action [141]. This foregrounds the ways in which animals explore, interact with and learn about their worlds, and the physiological mechanisms that mediate this; what Cisek & Pastor-Bernier [142] call the ‘challenges of embodied decision-making faced by animals interacting with their environment in real time’. An animal’s perceptual systems, its body plan and way of moving around the world guided by those senses (e.g. intricate hand movements guided by high-acuity stereoscopic vision, chemotactile exploration of a three-dimensional aquatic environment, sonar-guided movement through a multidimensional socio-ecological space) are all part and parcel of cognition. Similar to Cheng [143], who discusses a range of examples in some detail, Hodge *et al.* [110] who provide an exemplary integration of ecology, behaviour, sensory systems and cognition and Coombs & Trestman [96] who show, how across the metazoan tree of life, ‘brain and cognitive complexity can be attributed to the pivotal role played by body, sensory, brain and motor traits’, we encourage comparative psychologists to take this idea seriously and, instead of trying to isolate cognitive processes from the sensory-motor mechanisms that directly mediate animals’ behavioural interactions with their environments, more fully explore their interconnections.

## Data Availability

This article has no additional data.

## Footnotes

^1^
Associative learning (AL) is often posited as the low-level alternative to cognition. Detailed discussion is beyond the scope of this paper, but the editor has asked us to comment. In brief, we think the distinction between AL and cognition is erroneous and another stumbling block to progress in comparative psychology. As long ago as 1988, Rescorla [12] suggested that psychologists sometimes have an inaccurate and outmoded view of AL as incapable of efficiently producing intelligent behaviour. Through processes such as autoshaping, chaining and hierarchical organization of associations among associations, ‘conditioning involves the learning of relations among events that are complexly represented’. As Heyes put it more recently, ‘in most respects, contemporary associative learning theory is thoroughly cognitive’ [13]. AL can thus result in complex (multi-component, flexibly expressed, goal directed) behaviours, integrating multiple different sources of information. Hence, AL is neither a cognitive process nor an alternative to cognition: it is a developmental process by which the state of the animal and consequently its behavioural responses, are affected by experience, sometimes in complicated ways that enable it to solve problems and make ‘clever’ decisions. AL is therefore not only compatible with cognitive accounts of behaviour, it is not even in principle an alternative to them.

^2^
A striking illustration of the intractability of these debates is a report [30] in the journal *Science* 13 years ago. Many of the same arguments described then continue unabated today.

^3^
We can also point here to Moravec’s paradox that sensorimotor tasks that seem effortless to us, such as picking up a jug of water and pouring the water into a glass, require elaborate visuo-motor mechanisms and enormous neural resources, compared with tasks that we subjectively find more difficult, such as playing chess to a high level. The state of the art of robotics is still such that the water is likely to end up everywhere except in the glass. On the other hand, AI programmes like ‘Deep Blue’ have been capable of defeating chess grandmasters since the late 1990s. Subjective triviality and extensive neural resourcing are precisely what one would expect given that most of the 4 billion years of life on earth have been spent evolving and refining elaborate sensory-motor mechanisms for the direct guidance and control of fitness-enhancing behaviours [70].

^4^
Even then, researchers sometimes seem to forget to make such an adjustment. Maclean *et al.* [56], for example, attempted to quantify executive control across a phylogenetically diverse array of species using visuo-motor tasks for species with greatly differing visual acuity and where differences in ability to track hand movements have been shown to affect performance [30,79].

^5^
Lyon and Keijzer make some similar points to ours, advocating a ‘biogenic … approach that begins with biology’ [124, p 38] and emphasizes the continuity between sensory-motor and cognitive processes, and contrasting this with the anthropogenic approach of traditional cognitive explanation that takes humans as the starting point. We, however, caution against their term ‘basal cognition’, in case it encourages the view that ‘higher’ cognition is something else.

^6^
The ways in which stimuli that are not present influence behaviour (e.g. [132]) certainly raises important questions. It does not, however, mandate representational inferences, and need not imply the involvement of completely separate mechanisms from those used when the stimuli are present. Indeed, there is much evidence that perception involves memory inasmuch as it involves inference from past sensory experience [133,134], just one of the ways in which memory and perception are entangled [106].

^7^
Where complex motility involves organized, often rapid, multi-component movement sequences guided by sensory-motor feedback and coordination of multiple effectors.

^8^
Barron *et al.* [140] provide a general phylogenetic framework in terms of major transitions in cognitive evolution, and interestingly discuss neural implementation mechanisms. We differ from their approach, however, in as much as their view of the cognitive processes invokes abstract representations and is presented within a computational framework in which sensory information plays a secondary role to central computation.
